# Morphometric Evaluation of the Craniovertebral Junction Using Computed Tomography: A Sex‐Based Analysis of 500 Adults

**DOI:** 10.1155/bmri/2388226

**Published:** 2026-03-14

**Authors:** Nuri Fidan, Aymelek Cetin, Alptekin Tosun

**Affiliations:** ^1^ Department of Anatomy, Institute of Health Sciences, Inönü University, Malatya, Turkey, inonu.edu.tr; ^2^ Department of Anatomy, Faculty of Medicine, Inönü University, Malatya, Turkey, inonu.edu.tr; ^3^ Department of Radiology, Faculty of Medicine, Giresun University, Giresun, Turkey, giresun.edu.tr

**Keywords:** craniometric measurements, craniovertebral anomaly, craniovertebral junction (CVJ), occipitocervical, radiological measurement

## Abstract

**Background and Objective:**

The anatomy of the craniovertebral junction (CVJ) varies considerably across populations, yet comprehensive Turkish‐specific morphometric data remain limited. We aim to establish normative CVJ measurements in Turkish males and females using computed tomography (CT).

**Study Design:**

Retrospective morphological study.

**Materials and Methods:**

A retrospective analysis of CT images from 500 patients (250 females and 250 males, aged 25–40 years) was conducted between January and December 2022. CVJ measurements were obtained, and sex‐related differences were assessed.

**Results:**

The mean atlantodental interval was 1.45 ± 0.01 mm, posterior atlantodental interval 19.35 ± 0.09 mm, McGregor line 79.78 ± 0.21 mm, Chamberlain line 76.95 ± 1.21 mm, McRae line 36.27 ± 0.12 mm, Wachenheim clivus‐canal angle 154.65 ± 0.45^°^, sphenoid angle 121.15 ± 0.39^°^ (no sex difference, *p* = 0.083), Welcher basal angle 130.02 ± 0.28^°^ (higher in females, *p* < 0.001), basion‐axial interval 7.13 ± 0.07 mm (higher in males, *p* = 0.011), basion‐dental interval 5.32 ± 0.06 mm, and craniocervical tilt angle 123.94 ± 0.44^°^ (higher in males, *p* < 0.001).

**Conclusion:**

This study provides a comprehensive CT‐based analysis of CVJ measurements in Turkish adults, establishes normative morphometric values, and shows that most parameters exhibit sex‐based differences. These population‐ and sex‐specific reference data may be crucial for improving the accuracy of clinical assessments and surgical planning.

## 1. Introduction

The craniovertebral junction (CVJ) is a biomechanically complex transition zone between the skull and cervical vertebrae, composed of the os occipitale, atlas (C1), axis (C2), and their associated muscles, joints, and ligaments [1]. The CVJ supports the cranium, allows motion via the atlanto‐occipital and atlantoaxial joints, and protects the brainstem. Each anatomical element and its interactions are critical to the unique biomechanical properties of this region [2, 3].

Various congenital, hereditary, acquired, traumatic, neoplastic, and infectious conditions can disrupt the structural integrity of the CVJ, potentially leading to instability and impingement of neurovascular elements [4]. Among the most severe consequences is migration of the dens axis toward the foramen magnum, which can compress the brainstem and lead to life‐threatening outcomes such as arrhythmia, blood pressure instability, respiratory depression, or sudden death due to medulla oblongata involvement [5]. Compression of the anterior spinal artery may result in vertebrobasilar insufficiency, transient ischemic attacks, or other neurological deficits [6].

Surgical approaches to this region include anterior, posterior, and lateral approaches, each of which requires precise anatomical knowledge to ensure accurate diagnosis and effective intervention [7]. Accordingly, understanding the morphometric features of the CVJ is essential to facilitate diagnosis and perform therapeutic interventions [1, 3]. The necessary craniometric measurements can be obtained using direct radiography, magnetic resonance imaging (MRI), or computed tomography (CT). Although simple and accessible, radiography is two‐dimensional and may be limited by operator‐, device‐, or dosage‐dependent variations. MRI addresses most of these challenges but is better suited to the analysis of soft tissues and is expensive. Multidetector CT provides optimal visualization of bony structures with high resolution and minimal superimposition, making it the preferred method for detailed anatomical assessment of the CVJ [8, 9].

Despite numerous studies conducted worldwide, craniometric reference values may vary between populations due to racial, ethnic, and anatomical differences [10]. Importantly, growing evidence indicates that sex‐related differences affect craniometric parameters, highlighting the need for sex‐specific reference values to ensure accuracy in clinical assessments [11–13]. Although most existing CVJ studies rely on cadaveric specimens prone to shrinkage artifacts, sufficiently comprehensive radiologic data from living populations remain scarce [14, 15]. This is particularly true for the Turkish population, wherein comprehensive normative data, especially regarding population characteristics and sex‐related variations, are rare. This gap in sex‐stratified, population‐specific reference values may compromise diagnostic accuracy and surgical planning in clinical practice. Establishing these craniometric norms with consideration of ethnicity and biological sex is, therefore, crucial to improving the accuracy of radiologic assessments, which could enable early detection of pathologies and guide surgical interventions.

The aims of this study were to analyze craniometric parameters of the CVJ using CT in an adult Turkish population, to provide standardized reference values that could inform diagnostic and therapeutic processes, and to identify sex‐related differences.

## 2. Materials and Methods

### 2.1. Study Design and Setting

This retrospective, cross‐sectional, single‐center study was approved by the Ethics Committee for Clinical Research of Giresun Training and Research Hospital (Decision No. 10.07.2023/08) in accordance with relevant ethical principles and regulations, including the Declaration of Helsinki and Good Clinical Practice guidelines. Medical records of patients aged 25–40 years who presented to Giresun Training and Research Hospital, Giresun, Turkey, between January 2022 and December 2022 were reviewed. Data on age, sex, and CT images were obtained from the digital medical records system of our hospital.

### 2.2. Sample Selection

The study included 500 patients with intact CVJ anatomy who had no history of surgical intervention in this region, demonstrated no congenital or acquired anomalies, and had complete demographic and radiological records. To ensure balanced sex distribution and minimize sex‐related selection bias, the cohort comprised 250 randomly selected males and 250 randomly selected females. We excluded patients with any compromise of CVJ anatomical integrity, including those with prior surgical procedures involving this region, the presence of tumors or other pathological lesions, or artifacts that might interfere with craniometric measurements. Cases with incomplete data were also excluded from the analysis.

### 2.3. CT Imaging Protocol

All CT imaging was performed using a 128‐detector CT system (Revolution Evo 128, GE Healthcare, Milwaukee, Wisconsin, United States) with standardized acquisition parameters. The scanning protocol utilized 120‐kVp tube voltage and 127 effective mAs, with a rotation time of 0.6 s. Images were acquired with 1.25‐mm slice thickness and 1.2‐mm intervals and were reconstructed with 0.625‐mm slice thickness and intervals. All examinations were performed after obtaining proper localization on the scout image and in the supine position, with patients holding their breath to minimize motion artifacts. No oral or intravenous contrast material was administered. The acquired images were processed using a bone filter and bone window settings and were evaluated on a workstation by two investigators, who reached consensus decisions through joint review using the Akgün Picture Archiving and Communication System.

### 2.4. Acquisition of Craniometric Measures

Various parameters were measured in the midsagittal plane as follows:

#### 2.4.1. Atlantoaxial Measurements

The atlantodental interval (ADI) was measured as the distance between the anterior arch of C1 and the closest anterior point of the dens [16]. The posterior atlantodental interval (PADI) was measured as the distance from the posterior dens border to the closest point of the C1 posterior arch [16] (Figure 1).

Figure 1Midsagittal CT measurements of atlantodental intervals. (a) Atlantodental interval (ADI) shown as the red plus between the anterior arch of atlas (A) and dens axis (D), representing the normal space between C1 and the odontoid process. (b) Posterior atlantodental interval (PADI) shown as the red line between the posterior aspect of dens axis (D) and posterior arch of atlas (P), indicating the space available for the spinal cord at the C1–C2 level.

**Figure:**
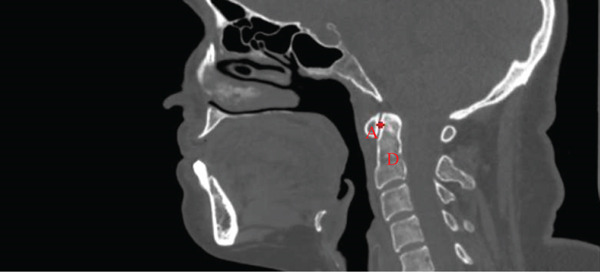
(a)

**Figure:**
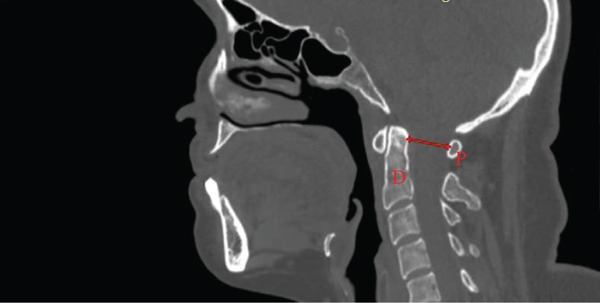
(b)

#### 2.4.2. Baseline Measurements

The McGregor line was measured as the line from the posterior hard palate to the most caudal point of the occipital bone [13]. The Chamberlain line was defined as the distance from the posterior hard palate to the opisthion [17]. The McRae line was measured as the line from the basion to the opisthion [11] (Figure 2).

Figure 2Midsagittal CT measurements of craniometric basilar lines. (a) McGregor line (red line) extends from the posterior aspect of the hard palate (P) to the most caudal point of the occipital bone (C) used to evaluate basilar invagination. (b) The Chamberlain line (red line) connects the posterior hard palate (P) to the opisthion (O), assessing for cranial settling. (c) The McRae line (red line) demarcates the foramen magnum between the basion (B) and opisthion (O), defining the functional diameter of the craniocervical junction.

**Figure:**
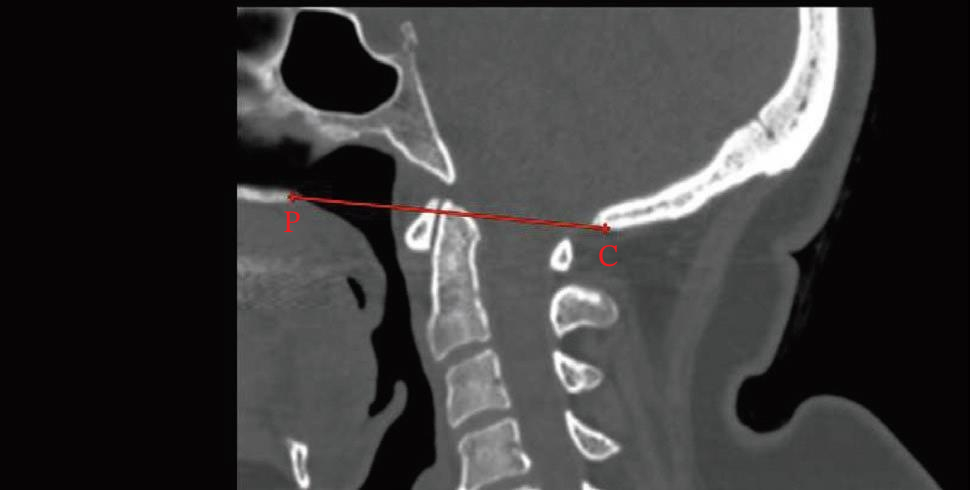
(a)

**Figure:**
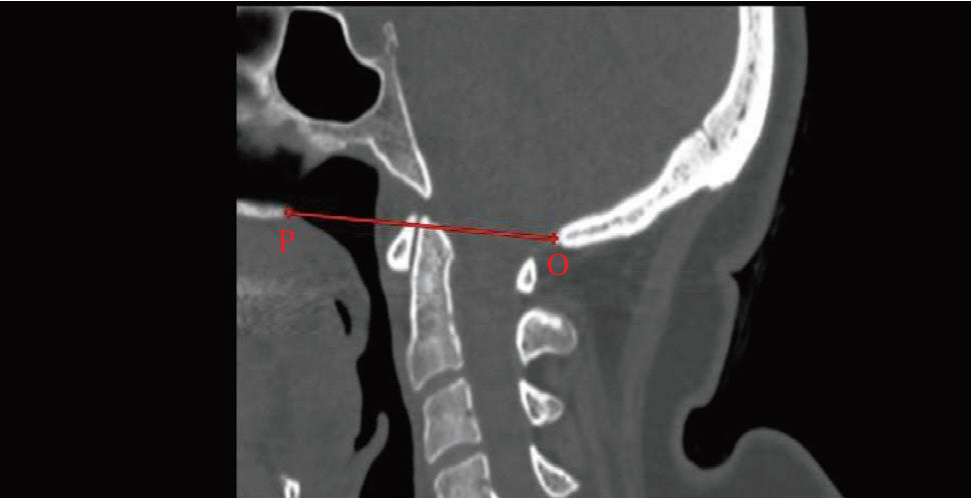
(b)

**Figure:**
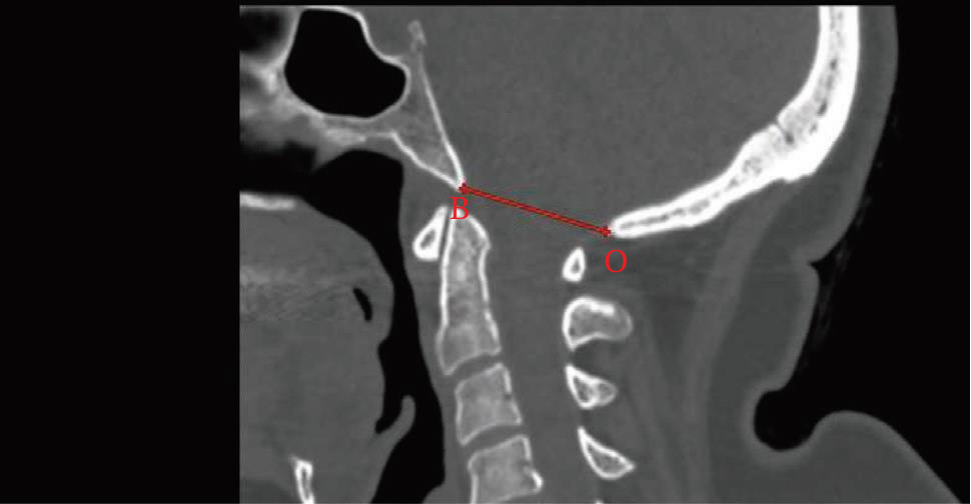
(c)

#### 2.4.3. Angular Measurements

**Figure 3:**
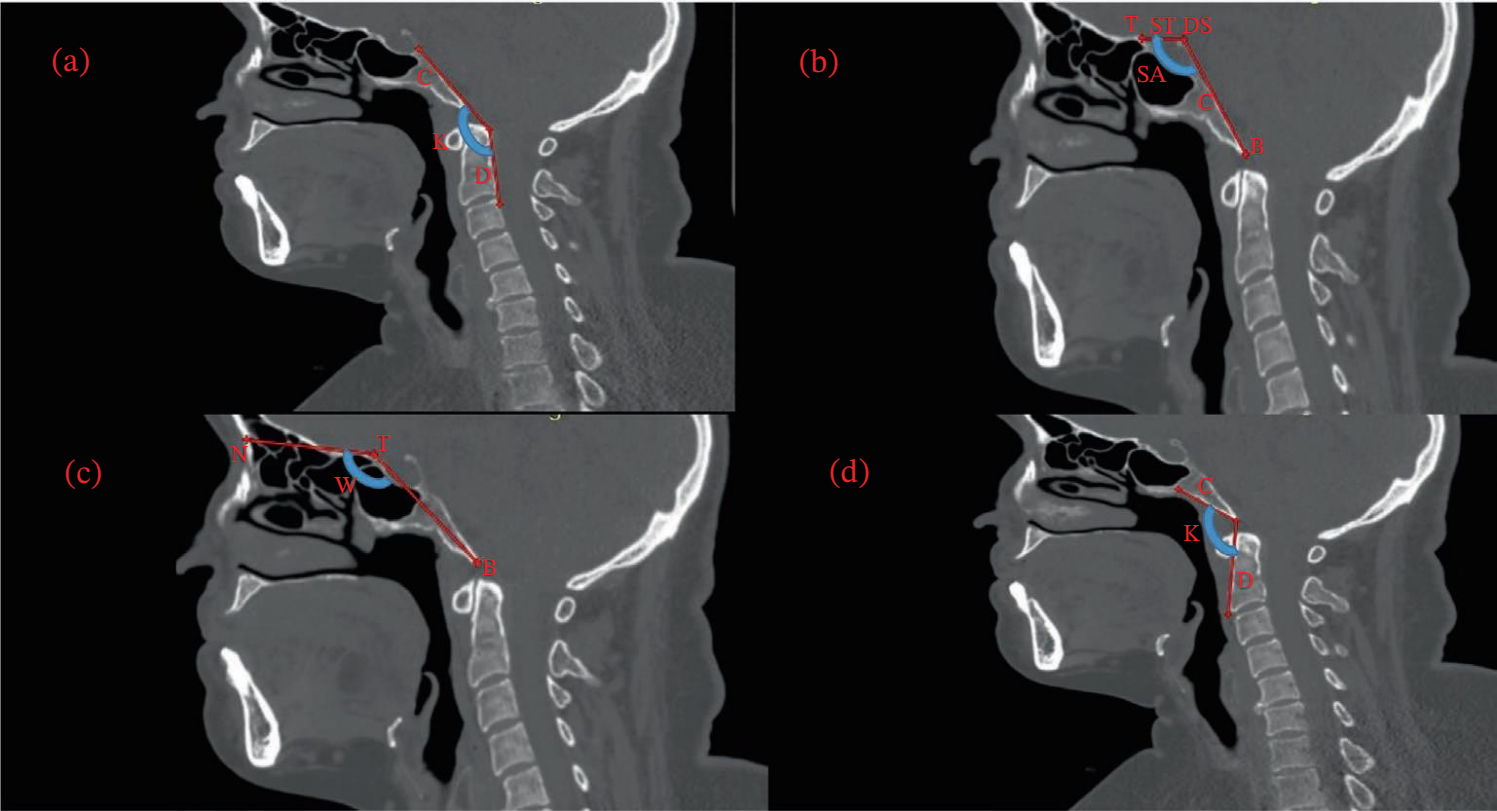
Midsagittal CT angular measurements of craniovertebral relationships.(a) Wachenheim clivus‐canal angle (K°, blue line) formed by lines along the posterior clivus (C) and posterior cervical canal at the dens (D), assessing brainstem compression risk. (b) Sphenoid angle (SA°, blue line) measured between the sphenoid base (ST), dorsum sellae (DS), and clivus (C), evaluating skull base flattening.(c) Welcher basal angle (W°, blue line) formed by basion (B)‐tuberculum sellae (T) and nasion (N)‐tuberculum sellae (T) lines, indicating platybasia when >140°.(d) CTA (K°, blue line) between anterior clivus (C) and dens axis (D), reflecting head‐neck alignment.

The Wackenheim clivus‐canal angle (CCA) was measured as the angle between the clivus and the cervical canal along the posterior surface of the dens [18]. The sphenoid angle was measured between the sphenoid base and the clivus [19]. The Welcher basal angle was defined as the angle between two lines passing from the tuberculum sellae: the basion‐tuberculum sellae line and the nasion‐tuberculum sellae line [13]. The craniocervical tilt angle (CTA) was measured as the angle between the anterior surface of the odontoid process and the anterior border of the clivus [7] (Figure 3).

#### 2.4.4. Basion‐Related Measurements

The basion‐axial interval (BAI) was measured as the distance from the basion to the extension of the posterior axis cortical line [13]. The basion‐dental interval (BDI) was defined as the distance from the basion to the superior point of the dens [20] (Figure 4).

Figure 4Midsagittal CT measurements of basion relationships. (a) The basion‐axial interval (BAI, green line) measures the distance from the basion (B) to the rostral extension of the posterior axial line (red line), assessing for vertical translocation. (b) The basion‐dental interval (BDI, red line) quantifies the distance between the basion (B) and the apex of the dens (A), evaluating superior migration of the odontoid process.

**Figure:**
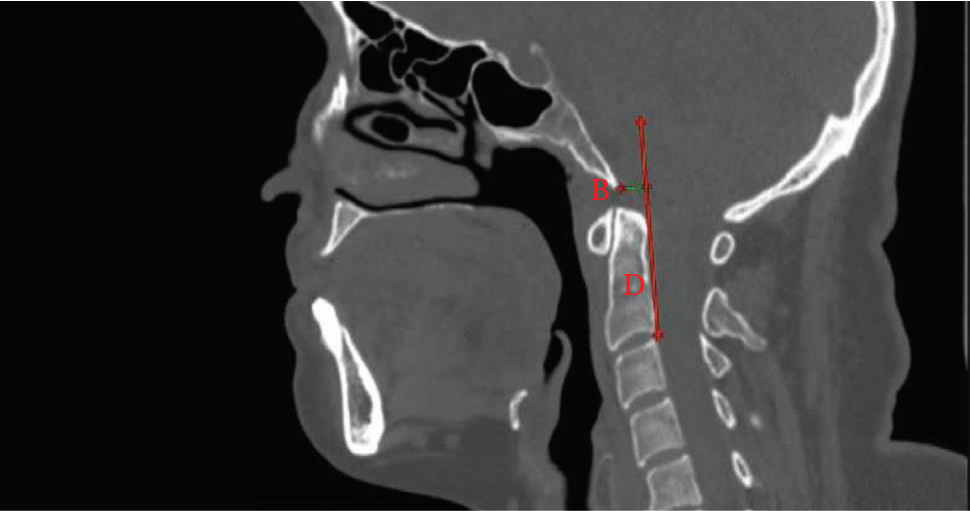
(a)

**Figure:**
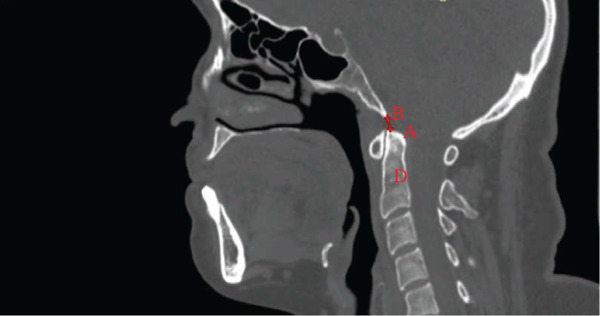
(b)

### 2.5. Statistical Analysis

The normality of quantitative data was assessed using the Kolmogorov–Smirnov test. Quantitative data that did not follow a normal distribution were summarized as median (minimum–maximum), whereas normally distributed quantitative data were expressed as mean ± standard deviation. For statistical comparisons between two independent groups, the independent samples *t*‐test was used for normally distributed variables, and the Mann–Whitney *U* test was applied for nonnormally distributed data. A *p* value of <0.05 was considered statistically significant. All analyses were performed using IBM SPSS Statistics 26.0 for Windows (New York, United States).

## 3. Results

All craniometric measurements, including comparisons between males and females, are presented in Table 1. Mean ADI was 1.45 ± 0.01 mm (median of 1.45 mm; range 0.36–2.92 mm). PADI averaged 19.35 ± 0.09 mm (median 19.31 mm; range 13.11–26.81 mm). The McGregor line measured 79.78 ± 0.21 mm (median 79.51 mm; range 65.79–94.23 mm), the Chamberlain line 76.95 ± 1.21 mm (median 76.58 mm; range 63.04–92.53 mm), and the McRae line 36.27 ± 0.12 mm (median 36.15 mm; range 28.53–43.11 mm). Angular measurements revealed a CCA of 154.65 ± 0.45^°^ (median 154.14°; range 116.03–179.62°), a sphenoid angle of 121.15 ± 0.39^°^ (median 120.91°; range 104.03–148.69°), a Welcher basal angle of 130.02 ± 0.28^°^ (median 130.04°; range 113.06–146.55°), and a CTA of 123.94 ± 0.44^°^ (median 124.04°; range 104.09–154.04°). The BAI averaged 7.13 ± 0.07 mm (median 7.14 mm; range 2.54–11.34 mm), and the BDI was 5.32 ± 0.06 mm (median 5.33 mm; range 1.66–9.34 mm).

**Table 1:** Craniometric measurement results of the patients.

Parameters	Total (*n* = 500)		Female (*n* = 250)		Male (*n* = 250)		*p*
	*M* *e* *a* *n* ± *S* *D*	Median (min–max)	*M* *e* *a* *n* ± *S* *D*	Median (min–max)	*M* *e* *a* *n* ± *S* *D*	Median (min–max)	
**ADI (mm)**	1.45 ± 0.01	1.45 (0.36–2.92)	1.54 ± 0.02	1.56 (0.58–2.49)	1.36 ± 0.02	1.335 (0.36–2.92)	**< 0.001** ^∗^
**PADI (mm)**	19.35 ± 0.09	19.31 (13.11–26.81)	18.83 ± 0.12	18.74 (13.11–24.51)	19.86 ± 0.13	20.03 (14.51–26.81)	**< 0.001** ^∗^
**McGregor line (mm)**	79.78 ± 0.21	79.51 (65.79–94.23)	78.41 ± 0.27	78.41 (65.79–89.73)	81.14 ± 0.31	81.055 (68.31–94.23)	**< 0.001** ^∗^
**Chamberlain line (mm)**	76.95 ± 1.21	76.58 (63.04–92.53)	75.37 ± 0.26	75.275 (63.04–85.6)	78.53 ± 0.30	78.61 (65.32–92.53)	**< 0.001** ^∗^
**McRae line (mm)**	36.27 ± 0.12	36.15 (28.53–43.11)	35.26 ± 0.13	35.34 (28.81–40.31)	37.28 ± 0.18	37.335 (28.53–43.11)	**< 0.001** ^∗^
**CCA (** ^**°**^ **)**	154.65 ± 0.45	154.14 (116.03–179.62)	155.77 ± 0.62	155.13 (116.03–179.62)	153.53 ± 0.66	153.055 (120.03–179.04)	**0.008** ^∗^
**Sphenoid angle (** ^**°**^ **)**	121.15 ± 0.39	120.91 (104.03–148.69)	121.82 ± 0.54	121.165 (104.11–148.69)	120.49 ± 0.56	120.035 (104.03–144.05)	0.083 ^∗∗^
**Welcher basal angle (** ^**°**^ **)**	130.02 ± 0.28	130.04 (113.06–146.55)	131.05 ± 0.42	131.075 (118.02–146.55)	129.00 ± 0.37	129.04 (113.06–145.06)	**< 0.001** ^∗∗^
**BAI (mm)**	7.13 ± 0.07	7.14 (2.54–11.34)	6.95 ± 0.10	6.945 (2.54–11.34)	7.32 ± 0.11	7.58 (3.23–10.31)	**0.011** ^∗∗^
**BDI (mm)**	5.32 ± 0.06	5.33 (1.66–9.34)	5.39 ± 0.09	5.41 (2.09–9.08)	5.24 ± 0.10	5.205 (1.66–9.34)	0.299 ^∗^
**CTA (** ^**°**^ **)**	123.94 ± 0.44	124.04 (104.09–154.04)	121.87 ± 0.50	122.98 (104.09–144.03)	126.01 ± 0.71	126.03 (105.04–154.04)	**< 0.001** ^∗∗^

*Note:* Data are given as mean ± standard deviation, median (minimum–maximum). Single asterisk denotes independent sample *t*‐test; double asterisks denote Mann–Whitney *U* test.

Abbreviations: ADI, atlantodental interval; BAI, basion‐axial interval; BDI, basion‐dental interval; CCA, clivus‐canal angle; CTA, craniocervical tilt angle; mm, millimeter; PADI, posterior atlantodental interval; SD, standard deviation.

Sex comparisons revealed significant differences in several parameters. Females had significantly higher values for ADI (*p* < 0.001), CCA (*p* = 0.008), and Welcher basal angle (*p* < 0.001), whereas males had significantly greater PADI (*p* < 0.001), McGregor line (*p* < 0.001), Chamberlain line (*p* < 0.001), McRae line (*p* < 0.001), BAI (*p* = 0.011), and CTA (*p* < 0.001). No significant sex differences were observed for the sphenoid angle (*p* = 0.083) or BDI (*p* = 0.299) (Table 1).

## 4. Discussion

This retrospective morphometric analysis of 500 adult CT scans provides a reliable basis for normative CVJ data and reveals multiple sex‐related differences. Females exhibited significantly higher values for ADI, CCA, and Welcher basal angle compared with males. Conversely, males had significantly greater PADI, McGregor line, Chamberlain line, McRae line, BAI, and CTA measurements. No significant sex differences were observed for sphenoid angle or BDI.

Pathologies of the CVJ typically progress insidiously and manifest in the late term but may also recur, albeit rarely [1, 3]. Common CVJ anomalies include basilar invagination, platybasia, atlas assimilation, and atlantoaxial dislocation, which can seriously affect critical neural structures such as the brainstem and spinal cord [4–6]. Radcliff et al. [21] reinforced the importance of radiological assessment in defining normal anatomic boundaries by sharing CT‐based normative data for the upper cervical spine. Craniometric measurements play a critical role in the diagnosis and treatment of these pathologies, and CT is considered the most advantageous imaging modality for their assessment [9]. Several studies have examined different populations and demonstrated variations that can be attributed to ethnic, methodological, and sex‐based differences. A comprehensive overview of available research on this topic is presented in Table 2. One important limitation in the literature is the scarcity of data from healthy populations, which is particularly evident in Turkey. Therefore, our aim was to perform CT‐based craniometric measurements of the CVJ in a large Turkish cohort.

**Table 2:** Comparative evaluation of craniovertebral junction measurements in varied populations.

Authors (year) [ref]	Study design and purpose	Population	Radiological method	Summary of findings
Our study	Retrospective cross‐sectional; normative CVJ craniometry by sex.	500 asymptomatic adults (250M/250F)	CT	ADI: 1.45 ± 0.01 mm (higher in females). PADI: 19.35 ± 0.09 mm (higher in males). McGregor line: 79.78 ± 0.21 mm (higher in males). Chamberlain line: 76.95 ± 1.21 mm (higher in males). McRae line: 36.27 ± 0.12 mm (higher in males). CCA: 154.65 ± 0.45^°^ (higher in females). Sphenoid angle: 121.15 ± 0.39^°^ (no sex difference). Welcher basal angle: 130.02 ± 0.28^°^ (higher in females). CTA: 123.94 ± 0.44^°^ (higher in males). BAI: 7.13 ± 0.07 mm (higher in males). BDI: 5.32 ± 0.06 mm (no sex difference).
Adam (1987) [19]	Radiographic norms versus basilar impression.	100 normals, 10 patients	X‐ray	Normal basal angle: 113^°^ ± 7^°^ versus 122^°^ ± 6^°^ in patients. Odontoid tip: 1.2 ± 2.28 mm below the McGregor line (normals) versus 9 ± 2.7 mm above (patients).
Al‐Dwairy et al. (2023) [17]	Retrospective; normative CVJ craniometry in asymptomatic patients.	137 asymptomatic adults (64M/73F)	CT	Chamberlain line length: 79.6 mm (no sex difference). No significant age or sex differences in other CVJ parameters were reported.
Alım et al. (2019) [28]	Prospective 3D CT morphometry of the odontoid process.	100 patients (55M, 45F; age‐stratified)	3D CT reconstruction	Odontoid width (OPmin): 11.3 mm (males > 50 yrs). Odontoid + vertebral body AP (OPAP): 14.5 mm (males > 50 yrs). LAIT (length to dens tip): 45.2 mm (males > 50 yrs). All metrics are higher in males versus females. A single 4.5‐mm screw is feasible in most cases.
Bakirci et al. (2014) [47]	Morphometric analysis of Byzantine‐era C2 vertebrae	70 excavated vertebrae	Photogrammetry (Scion Image)	Superior facet distance: 42.7 ± 0.5 mm. Facet angle: ~115° bilaterally. Corpus width (inferior): 22.7 ± 0.3 mm. Corpus area:38.7 ± 1.1 mm^2^. Symmetry in facet‐dens distances (23–24 mm lateral, 12–13 mm medial).
Batista et al. (2015) [22]	Cross‐sectional; normative CT‐based CVJ craniometry in asymptomatic adults.	100 asymptomatic adults (52M/48F)	CT	ADI: 1.1 mm (mean). CCA: 153.68° (range: 132.32°–173.95°). Basal angle: 113.73° (range: 97.06°–133.26°). Odontoid position: 5% > 2 mm above Chamberlain line; 1% >5 mm. Clivus length: 44.74 mm. External occipital protuberance thickness: 7.42–22.36 mm. No sex differences reported for main parameters.
Bosco et al. (2018) [43]	CT morphometry for occipital condyle screw fixation.	70 Indian adults (35M/35F)	CT	OC screw feasibility: 73% population. Safe screw length = 19.9 ± 2.3 mm. Angulations: ≤ 6.4° cranial, 31.1° medial. C0–C1 transarticular screws: Length = 26.7–31.6 mm, angles = 36.7°–48.9°. 27% unsuitable for OC screws due to anatomy. Vertebral artery injury risk is high with the caudal C1 arch technique.
Chen et al. (2011) [23]	Cross‐sectional; normative AADI/LADI values in asymptomatic Chinese adults.	230 asymptomatic adults (140M/90F)	Reformatted CT	AADI: 1.83 ± 0.46 mm (males), 1.63 ± 0.43 mm (females) (*p* < 0.05). LADI (left/right): 3.38 ± 0.87 mm (M), 3.30 ± 0.73 mm (F) (no sex difference). LADI asymmetry: 0.76 ± 0.66 mm (M), 0.73 ± 0.70 mm (F) (normal variant). 95% CI for AADI: 1.75–1.90 mm (M), 1.54–1.72 mm (F).
Dash et al. (2018) [24]	Retrospective: CVJ osteometry in asymptomatic Indian adults.	49 asymptomatic adults (31M/18F)	CT angiogram	ADI: < 2.5 mm in all patients. Odontoid tip position: Mean 5.11 mm below McRae line (none above). Sex differences: Smaller C1 lateral masses and odontoid screw trajectory in females (*p* < 0.05). Other parameters (e.g., PADI) similar to global data.
Frade et al. (2017) [38]	Cross‐sectional; platybasia parameters in NE Brazil.	181 asymptomatic adults	MRI	Welcker basal angle: 128.96^°^ ± 6.51^°^. Clivus‐canal angle: 150.5° (IQR 143.2–157.3). Inverse correlation between basal and clivus‐canal angles (*p* < 0.05).
Gonzalez et al. (2004) [20]	Radiologic criteria for C1–2 distraction.	93 controls, 5 patients.	CT/MRI	Normal BDI: 4.7 ± 1.7 mm (95% CI 0.6–9 mm). LMI > 2.6 mm suggests injury (normal: 1.7 ± 0.48 mm). MR signal increase confirms injury.
Gosavi et al. (2012) [29]	Dry bone morphometry of atlas vertebrae	100 Indian atlases	Digital vernier caliper	Atlas width (TD): 69.37 mm. Superior facet shape: 74% oval, 26% kidney‐shaped. Inferior facet shape: 71% circular. Vertebral artery groove thickness: 3.7 mm bilaterally. No significant side differences.
Gupta et al. (2020) [16]	Retrospective; normative CVJ data in rural Indian adults.	255 asymptomatic adults (175 M/80F)	CT	AADI: No sex difference. PADI, clivus length, foramen magnum diameter, and Boogard′s/basal angles: Significant sex differences (*p* < 0.05). Age differences: ADI, clivus length, and foramen magnum diameter varied significantly between ≤ 20 and > 20 years (*p* < 0.05).
Erçakmak B. and vatansever A. (2018) [3]	Retrospective morphometric analysis of FM and clivus	313 Turkish adults (165M/148F)	CT angiography	Clivus length (LoC): 32.1 ± 3.4 mm (F), 35.2 ± 4 mm (M). FM AP diameter (FMap): 34.72 ± 2.57 mm (F), 36.29 ± 2.89 mm (M). FM area (FMa): 733.26 ± 102.09 mm^2^ (F), 800.12 ± 110.62 mm^2^ (M). All metrics are larger in males (*p* < 0.05).
Harris et al. (1994) [46]	Normal occipitovertebral relationships.	400 adults, 50 children.	X‐ray	Normal BAI ≤ 12 mm (98% adults). BDI ≤ 12 mm (adults), unreliable in children. Flexion/extension BAI excursion ≤ 10 mm.
Henderson et al. (2018) [39]	Prospective pilot; clivo‐axial angle (CXA) and brainstem deformity.	10 adults with pathological CXA; pre/post‐op fusion.	MRI/CT	Mean CXA improved from 135.8° to 163.7° post‐op. Significant clinical improvement correlated with CXA normalization (*p* < 0.05). CXA < 135° suggests brainstem risk.
Ilhan et al. (2017) [14]	Dry bone FM/condyle morphometry	100 Turkish skulls	Digital caliper	FM dimensions: AP = 35.17 ± 2.94 mm, transverse = 29.73 ± 2.53 mm. Condyle dimensions: AP = 23.47 ± 2.44 mm, width = 11.40 ± 1.41 mm. Intercondylar distances: anterior = 22.47 ± 2.98 mm, posterior = 41.54 ± 3.78 mm. FM shapes: 24% tetragonal (most common). Side differences in condyle‐opisthion distances (*p* < 0.05).
İnce et al. (2021) [15]	Dry bone morphometry of CVJ components	9 occipital bones, 18 atlases, 16 axes	Digital caliper	Atlas: All transverse measurements (processes/foramina) are shorter in females (*p* < 0.05). Vertebral canal AP/transverse diameters: males > females (*p* = 0.014). Axis: Dens width (9.03 ± 0.57 mm F vs. 10.61 ± 10.54 mm M, *p* < 0.001), corpus height (22.6 ± 1.47 mm F vs. 24.62 ± 0.85 mm M, *p* = 0.034). Occipital: FM AP = 34.2 ± 2.8 mm, transverse = 29.35 ± 3.17 mm. Condyle lengths: 23.65 mm (R), 24.58 mm (L).
Koenigsberg et al. (2005) [40]	Retrospective; MR platybasia evaluation.	250 subjects (200 adults, 50 children).	MRI	Standard basal angle: 129^°^ ± 6^°^ (adults), 127^°^ ± 5^°^ (children). Modified technique: 117^°^ ± 6^°^ (adults), 114^°^ ± 5^°^ (children) (*p* < 0.05 vs. radiographs).
Liu et al. (2015) [27]	Cross‐sectional; age‐stratified ADI reference ranges in Chinese adults.	700 asymptomatic adults (7 age groups: 18–24 to >70 years)	MDCT	ADI: Decreased linearly with age (*r* = −0.511, *p* = 0.001). Mean ADI by age group: 1.77 mm (18–24), 1.61 mm (25–29), 1.58 mm (30–39), 1.41 mm (40–49), 1.31 mm (50–59), 1.34 mm (60–69), and 1.06 mm (> 70). No sex difference (*p* = 1.000). Proposed age‐specific reference ranges: 0.85–3.12 mm (18–39), 0.71–2.55 mm (40–59), and 0.00–2.37 mm (> 60).
Lyrtzis et al. (2017) [11]	Dry bone FM/condyle/hypoglossal canal analysis	141 Greek skulls (73M,68F)	Digital caliper	Sex differences: FM AP (M = 36.16 ± 2.29 mm, F = 33.86 ± 2.31 mm), condyle length (M = 24.2 ± 2.58 mm, F = 23.09 ± 2.84 mm). HC variants: 24.1% long HCs (> 7.37 mm R, > 7.59 mm L), 27.7% short condyles (< 21.80 mm). Age impact: FM AP decreased from 36.41 mm (young) to 34.75 mm (elderly). Septated HCs: 23.6%.
Marathe et al. (2019) [32]	Retrospective; morphometric analysis of skull baselines (Chamberlain, McGregor, and McRae lines) in Indians	116 asymptomatic Indian patients	X‐ray and CT scan	Chamberlain line: 55.12% had odontoid tip above line; mean distance: CT = 0.498 mm, x‐ray = 0.528 mm. McGregor Line: 58.62% had odontoid tip above line; mean distance: CT = 0.213 mm, x‐ray = 0.228 mm. McRae Line: Mean distance: CT = 4.67 ± 1.69 mm, x‐ray = 4.7 ± 1.76 mm; no cases crossed the McRae line. Sex differences: No significant differences in males versus females. Imaging comparison: No significant difference between x‐ray and CT measurements. The McRae line recommended for screening.
Nalbant et al. (2024) [31]	Retrospective; sex/age‐based CVJ anatomy in Anatolian adults.	100 asymptomatic adults (50M/50F), age 25–45 years	CT	Sex differences: Lower values in females for cervical lordosis angle, ADI (1.58 ± 0.53 mm), PADI (19.33 ± 2.87 mm), McRae line (4.89 ± 1.54 mm), CCA (155.57 ± 8.09^°^), clivus length (36.76 ± 5.39 mm), and dens axis dimensions (*p* < 0.05). Power ratio decreased in age < 30 (*p* < 0.05). No age effect on ADI/PADI.
Netto et al. (2014) [41]	Metric analysis of basal sphenoid angle.	160 adult skulls.	Physical measurement	Mean basal angle: 115.41^°^ ± 8.45^°^. No sex/age correlation. Higher in brachycephalic skulls (118.13° vs. 112.16° dolichocephalic, *p* < 0.05).
Omercikoglu et al. (2017) [12]	Retrospective; age/sex‐based normative CT values for cervical metrics in trauma patients.	500 adult trauma patients (346M/154F)	MDCT	ADI: Wider in males; decreased with age in both sexes (*p* < 0.05). BDI: Wider in males; decreased with age in females (*p* < 0.05). Proposed upper limits: ADI ≤ 2.5 mm, BDI ≤ 8.5 mm
Osmotherly et al. (2012) [30]	Prospective; MRI evaluation of stress tests (anterior shear/distraction) in asymptomatic volunteers.	16 asymptomatic adults (8M/8F), age 19–32 years	MRI (3‐T)	ADI (neutral): 2.29 ± 0.47 mm; increased to 2.70 ± 0.57 mm during anterior shear test (+0.41 mm, *p* = 0.03). BDI (neutral): 7.05 ± 2.28 mm; increased to 7.69 ± 2.45 mm during the distraction test (+0.64 mm, *p* < 0.01). Tectorial membrane length increased by 1.12 mm (*p* = 0.02). Reliable interobserver performance.
Radcliff et al. (2010) [21]	Retrospective anatomical review; define normal CT metrics for the upper cervical spine	76 trauma patients (no cervical injury)	High‐speed CT	OCI: ≤ 1.0 mm (95% CI). AA joints: Coronal plane more reproducible (95% CI: 1.2 mm). Dens alignment: Symmetrical (side‐to‐side difference ≤ 1.6 mm). No age/race/sex differences. Nonnormal distribution for most parameters.
Singla et al. (2015) [48]	Anatomical study of Indian axis vertebrae	30 dry axis specimens	Digital caliper/mini‐inclinometer	Odontoid AP diameter: 10.1 mm. Transverse diameter: 9.32 mm. Pedicle width: 10.07–10.52 mm. Vertebral canal inlet AP: 18.31 ± 2.05 mm. Dens angle: 13.23°. SAF: 84% oval, 16% circular.
Tanrisever et al. (2020) [13]	Retrospective; CBCT‐based CVJ morphometry in Turkish adults.	300 asymptomatic adults (145F/155M)	Cone‐beam CT	ADI: Decreased with age (*r* = −0.517, *p* = 0.001). BAI: Decreased with age (*r* = −0.217, *p* = 0.007). Sex differences: Significant in lateral mass‐related parameters (*p* < 0.05). Odontoid position: 36% above McGregor line, 28.9% above Chamberlain line. Basion position: Anterior to axial line in 96.3%.
Tassanawipas et al. (2005) [33]	Retrospective; validation of basilar impression lines in the Asian population.	114 asymptomatic Thai adults (61M/53F)	MRI	Odontoid above Chamberlain′s line: 34% (mean 2.89 mm); 10.5% > 5 mm. McRae′s line: No cases above foramen magnum. Ranawat′s line: 15.75 mm (M), 14.09 mm (F). McGregor′s line: Odontoid extended 0.33 mm (M), 0.17 mm (F) above.
Thintharua et al. (2023) [44]	Morphometric analysis of the occipital condyle (OC) and its relation to surrounding structures	200 dry skulls	Anatomical dissection and measurements	OC morphometry: Mean length: 21.3 ± 2.4 mm, width: 10.5 ± 1.4 mm, height: 7.4 ± 1.1 mm. Distances: Anterior OC tip to basion: 11.5 ± 1.4 mm; posterior OC tip to basion: 39.1 ± 3.3 mm; anterior OC tip to opisthion: 25.2 ± 2.2 mm; posterior OC tip to opisthion: 27.4 ± 2.7 mm. Relationships: The hypoglossal canal (HC) orifice is most common is the most common in the middle 1/3 of the OC (45%); the jugular foramen (JF) is related to the anterior 2/3 of the OC (81%). No sex/age differences reported.

Abbreviations: AADI, anterior atlantodental interval; ADI, atlantodental interval; AP, anteroposterior; BAI, basion‐axial interval; BDI, basion‐dental interval; CBCT, cone‐beam computed tomography; CCA, clivus‐canal angle; CT, computed tomography; CTA, clivo‐temporal angle; CVJ, craniovertebral junction; F, female; FM, foramen magnum; HC, hypoglossal canal; JF, jugular foramen; LADI, lateral atlantodental interval; LAIT, length of axis including tip; LMI, lateral mass interval; M, male; MDCT, multidetector computed tomography; MRI, magnetic resonance imaging; OC, occipital condyle; OCI, occipital condyle interval; OPAP, odontoid process anteroposterior; OPmin, minimum odontoid process width; PADI, posterior atlantodental interval; SAF, superior articular facet; SD, standard deviation; TD, transverse diameter.

The ADI is a critical metric for diagnosing atlantoaxial instability, with normal values ≤ 3.5 mm in adults and values > 10 mm indicating rupture of the ligamentum transversum [22]. Although the atlantoaxial joint is stabilized by ligaments (lig. transversum, alaria, and atlantodental), an enlarged ADI suggests CVJ ligament injury [22]. Available data from healthy controls in previous studies report mean ADI values of 1.10–2.29 mm [12, 23–26], with substantial demographic variation. Batista et al. [22] found a mean ADI of 1.1 mm with no sex differences, whereas Chen et al. [23] reported higher ADI in males (1.83 mm vs. 1.63 mm, *p* < 0.05). Liu et al. [27] noted an age‐dependent decline (1.77 mm in 18–24‐year‐olds vs. 1.06 mm in > 70‐year‐olds), regardless of sex. Alım et al. [28] showed that odontoid process morphology can vary among populations and is of critical importance in preoperative planning, supporting the relevance of dens‐related measurements in our study. Gupta et al. [16] and Tanrisever et al. [13] observed no significant sex differences (1.32 mm vs. 1.25 mm and 1.29 mm vs. 1.27 mm, respectively), whereas Ömercikoglu et al. [12] reported higher ADI in males (1.81 mm vs. 1.66 mm) with age‐related decreases. Our study largely aligns with the literature in terms of absolute values (mean: 1.45 mm) but, interestingly, shows higher ADI in females (1.54 mm vs. 1.36 mm; *p* < 0.05), likely reflecting population‐specific anatomical variations [12, 13].

In CVJ assessment, PADI is also considered valuable for both anatomical and clinical purposes [16]. Gosavi et al. [29] reported significant variations in the anatomical structure of the atlas vertebra, which may affect CVJ measurements and their interpretation. Although Forseen and Borden [25] established > 14 mm as the reference threshold in adults (via radiography), McRae [26] noted that values < 19 mm may correlate with neurological symptoms, suggesting that a higher threshold may be more appropriate for defining reference values. Reported PADI measurements vary according to the imaging modality and population studied: Osmotherly et al. [30] found a mean of 11.89 ± 1.54 mm in MRI studies, whereas Gupta et al. [16] reported a broader range (12.10–26.20 mm; mean 16.77 mm), with significantly higher values in males (17.03 mm vs. 16.2 mm in females). Nalbant et al. [31] reported similar sex differences (males: 19.33 ± 2.87 mm vs. females: 17.84 ± 1.57 mm), as did Tanrisever et al. [13] (males: 20.20 mm vs. females: 18.82 mm). Notably, Dash et al. [24] found no sex disparity in PADI, although they confirmed smaller C1 lateral masses in females, which they suggested could affect surgical planning. Our results are consistent with the broader literature, showing a mean PADI of 19.35 mm overall, with males (19.86 mm) having significantly higher values than females (18.83 mm), aligning with the morphometric data provided by İnce et al. [15] regarding the vertebral canal space. Batista et al. [22] further emphasized comparable PADI values between sexes in some populations, highlighting the need for population‐specific normative data.

McGregor′s line, defined in 1948, is used to assess basilar invagination by evaluating the position of the odontoid process [13]. Although most studies focus on this relationship, the length of this line has rarely been examined. Tanrisever et al. [13] reported a mean length of 80.07 mm, with men (81.38 mm) having significantly higher values than women (78.79 mm). Similarly, our study found a mean of 79.78 mm, with men (81.14 mm) showing significantly greater line length than women (78.41 mm). Other studies reporting related data also support ethnic and racial variation in these measures. Marathe et al. [32] found that 58.62% of their Indian cohort had the odontoid tip above McGregor′s line (mean distance: 0.213 mm on CT vs. 0.228 mm on x‐ray), with no significant sex differences. Tassanawipas et al. [33] reported that the odontoid extended 0.33 mm above McGregor′s line in males versus 0.17 mm in females in their Thai population, suggesting a potential ethnic variation in these relationships.

First described in 1939, Chamberlain′s line is used to aid in the diagnosis of basilar invagination when the odontoid apex projects above this landmark. Al‐Dwairy et al. [17] measured its length as 79.62 mm, with no significant sex difference. Tanrisever et al. [13] reported a mean of 76.05 mm, with higher values in men (77.84 mm vs. 74.15 mm). Our results were similar: 76.95 mm overall, with men (78.53 mm) showing significantly higher values than women (75.37 mm). Marathe et al. [32] observed that 55.12% of their Indian subjects had the odontoid tip above Chamberlain′s line (mean distance: 0.498 mm on CT vs. 0.528 mm on x‐ray). Tassanawipas et al. [33] reported a high prevalence in their Thai cohort, with 34% having the odontoid above this line (mean 2.89 mm). Notably, 10.5% of their population had a difference exceeding 5 mm, demonstrating substantial variation both across and within populations.

McRae′s line, which represents the anteroposterior diameter of the foramen magnum, typically exceeds 30 mm in asymptomatic individuals [26]. Values below 25 mm often correlate with neurological symptoms [1, 26]. Dash et al. [24] found larger measurements in men than in women (36.48 mm vs. 35.97 mm). Tanrisever et al. [13] reported a mean of 35.58 ± 2.52 mm, with men (36.46 mm) having greater values than women (34.64 mm). Our findings were consistent with these reports, with an overall mean of 36.27 ± 0.12 mm and significantly higher values in men (37.28 mm) than in women (35.26 mm). Marathe et al. [32] reported mean McRae line distances of 4.67 ± 1.69 mm (CT) and 4.7 ± 1.76 mm (x‐ray) in their Indian population, with no cases in which the odontoid crossed this line. Similarly, Tassanawipas et al. [33] found no cases above McRae′s line in their MRI study of 114 Thai adults, confirming its reliability as a diagnostic threshold across populations. Nalbant et al. [31] also reported sex differences in McRae line‐related measurements. Furthermore, Erçakmak and Vatansever [3], Lyrtzis et al. [11], and Ilhan et al. [14] all identified significant sex‐based variations in foramen magnum dimensions, which are in strong agreement with the findings of our study.

The CCA, first described by Bundschuh et al. [34], normally ranges between 135° and 175°. Values below 135° may indicate myelopathy, and Smoker [18] noted that values under 150° can cause ventral spinal cord compression. Henderson et al. [35] linked narrow angles to brainstem deformation in traumatic or degenerative conditions, whereas Wang et al. [36] reported that higher angles correlate with better surgical outcomes in basilar compression. Data from asymptomatic individuals are generally consistent with these observations, with mean values ranging from 135° to 159.61°. Bundschuh et al. [34] reported a mean of 155.2°, with lower values in patients with rheumatoid arthritis (135°). Botelho and Ferreira [37] found 148^°^ ± 9.8^°^, and Nalbant et al. [31] reported 155.57^°^ ± 8.09^°^. Tanrisever et al. [13] observed 157.62^°^ ± 11.85^°^ with no sex differences. Our measurements were similar but showed a significant sex difference: an overall CCA of 154.65^°^ ± 0.45^°^, with women (155.77°) having a significantly higher angle than men (153.53°). Frade et al. [38] reported a median CCA of 150.5° (IQR 143.2–157.3) in their Brazilian cohort, demonstrating an inverse correlation with basal angle (*p* < 0.05). Henderson et al. [39] found that CCA increased from 135.8° to 163.7° postoperatively in patients undergoing surgery for ventral brainstem compression, and this change correlated with neurological improvement. Nalbant et al. [31] reported similar values (155.57 ± 8.09^°^) in a Turkish population.

The sphenoid angle, used to assess skull base development, typically ranges from 125° to 143° in adults [40, 41]. Values > 143° indicate abnormal cranial base shapes, whereas values < 125° have been proposed to suggest basilar kyphosis [40, 41]. Our study found a mean of 121.15° in healthy subjects, which interestingly falls within the proposed definition of “kyphosis.” There were no sex differences (men: 120.49°, women: 121.82°). Marked variation in reported values appears to be influenced by race and ethnicity: 113° in Kenya [19] and 129° in the United States [42]. Netto et al. [41] supported racial variation, reporting a mean basal angle of 115.41°, with brachycephalic skulls showing higher angles (118.13°) than dolichocephalic skulls (112.16°). Koenigsberg et al. [40] noted modified basal angles of 117^°^ ± 6^°^ in adults, differing from radiographic standards (129^°^ ± 6^°^), indicating that methodological factors, as well as race and ethnicity, may significantly affect sphenoid angle measurements. Taken together, these findings suggest that available data on this parameter and the definitions derived from them should be updated in light of new, population‐based evidence. Bosco et al. [43] highlighted the importance of condylar measurements in CVJ surgery by demonstrating that occipital condyle morphology significantly influences screw orientation and fixation security. Thintharua and Chentanez [44] showed that substantial anatomical variation exists between the occipital condyle, foramen magnum, and hypoglossal canal, with direct implications for CVJ surgical approaches.

Smoker [18] noted that the normal Welcher basal angle is approximately 132° and should not exceed 140°, suggesting that higher values are associated with skull base abnormalities. Values reported in asymptomatic populations range from 113.7° to 131.2°. Gupta et al. [16] found that women (131.4°) had significantly higher values than men (129.7°), whereas Tanrisever et al. [13] observed no sex difference (women: 131.25°, men: 130.40°). Our results aligned with those of Gupta et al., with a mean of 130.02° and significantly higher values in women (131.05°) than in men (129.00°). Frade et al. [38] documented a mean Welcker basal angle of 128.96^°^ ± 6.51^°^ in Brazilians, whereas Koenigsberg et al. [40] reported 129^°^ ± 6^°^ in adults in the United States (MRI‐based). Adam [19] found that pathological cases had a mean of 122^°^ ± 6^°^, compared with 113^°^ ± 7^°^ in healthy controls, reinforcing the association between elevated values and pathology.

The BAI, which is critical for diagnosing atlanto‐occipital dislocation, should not exceed 12 mm. Chang et al. [45] reported values of 3.90 mm in controls versus 8.11 mm in patients with blunt trauma. Tanrisever et al. [13] found a mean of 4.01 ± 1.83 mm with no sex differences. Conversely, our study recorded higher values (7.13 ± 0.07 mm), with men (7.32 mm) showing significantly higher measurements than women (6.95 mm). Harris et al. [46] established BAI ≤ 12 mm as normal (in 98% of adults) with flexion‐extension excursions ≤ 10 mm. Tanrisever et al. [13] reported that BAI decreased with age (*r* = −0.217, *p* = 0.007), suggesting dynamic changes over the lifespan, whereas Nalbant et al. [31] found no age effect on BAI‐related metrics.

Introduced by Wholey et al. [42], the BDI is used to detect cervical anomalies. Gonzalez et al. [20] reported values of 4.7 mm in controls versus 11.9 mm in patients with injury. Tanrisever et al. [13] reported a mean BDI of 4.92 mm with no sex difference. Our mean value was 5.32 mm (women: 5.39 mm, men: 5.24 mm), which is consistent with the literature and shows no sex disparity. Omercikoglu et al. [12] proposed BDI ≤ 8.5 mm as an upper limit in trauma patients, noting wider intervals in males and age‐related decreases in females. Gonzalez et al. [20] reported normal BDI values of 4.7 ± 1.7 mm (95% CI: 0.6–9 mm), with values > 9 mm suggesting injury. Bakirci et al. [47] reported substantial individual variation in C2 vertebral morphology and emphasized the clinical importance of population‐specific morphometric reference values for the upper cervical region. Singla et al. [48] similarly highlighted that morphometric variations in the axis vertebra have important implications for surgical stabilization, which is consistent with our findings related to C2.

Chandra et al. [49] linked CTA (84.0^°^ ± 15.10^°^) to basilar invagination versus controls (110.30^°^ ± 4.23^°^). Tanrisever et al. [13] found a mean CTA of 126.98° in asymptomatic individuals, with higher values in men (128.83°) than in women (125.01°). We observed an overall mean of 123.94°, with men (126.01°) having significantly larger angles than women (121.87°), supporting previously reported sex‐based differences [13, 34]. Batista et al. [22] reported a normative CTA of 153.68° (range: 132.32°–173.95°), whereas Frade et al. [38] noted an inverse correlation between basal and CCAs.

## 5. Conclusion

Although this study provides comprehensive morphometric data for CVJ parameters in a large cohort, several limitations should be acknowledged. First, the retrospective design may introduce selection bias, as our sample was drawn from patients who underwent cervical CT scans and may not represent the general population. Second, as illustrated by the variations observed when comparing our results with Asian and Western cohorts, these measures are strongly influenced by race, ethnicity, and potentially other environmental or genetic factors. Third, the study included only static CT analyses, without dynamic positioning or functional assessment. Fourth, although we identified significant sex‐based differences, the clinical relevance of these small but statistically significant variations requires further investigation. Finally, the study did not account for potential age‐related changes in CVJ anatomy beyond adulthood, as our analysis was restricted to young adults.

We provide comprehensive morphometric data on the CVJ in 500 adults from Turkey, revealing significant sex‐based differences: females had higher ADI (1.54 vs. 1.36 mm), CCA (155.77° vs. 153.53°), and Welcher angle (131.05° vs. 129.00°), whereas males exhibited greater PADI (19.86 vs. 18.83 mm), McGregor line (81.14 vs. 78.41 mm), Chamberlain line (78.53 vs. 75.37 mm), McRae line (37.28 vs. 35.26 mm), BAI (7.32 vs. 6.95 mm), and CTA (126.01° vs. 121.87°). Taken together with the variations reported in the literature and across sexes, these findings clearly demonstrate the need for sex‐ and population‐specific normative data to underpin CVJ evaluation. The CT‐based measurements presented here offer reliable, clinically applicable reference values that can enhance diagnostic precision and inform surgical planning in CVJ‐related conditions. Future multicenter studies are warranted to broaden and validate these normative datasets.

## Author Contributions

All authors contributed to the study conception and design. Material preparation, data collection, and analysis were performed by Nuri Fidan and Aymelek Cetin. The first draft of the manuscript was written by Nuri Fidan, and all authors commented on previous versions of the manuscript.

## Funding

No funding was received for this manuscript.

## Disclosure

All authors read and approved the final manuscript.

## Conflicts of Interest

The authors declare no conflicts of interest.

## Data Availability

The data that support the findings of this study are available on request from the corresponding author. The data are not publicly available due to privacy or ethical restrictions.
